# Expression of endogenous retroviral elements is associated with extracellular matrix remodeling in prostate cancer

**DOI:** 10.1186/s13100-025-00382-9

**Published:** 2026-01-08

**Authors:** Emily C. Williams, Dewanga R. Mayarata, Anelia Horvath, Katherine B. Chiappinelli, Maho Shibata

**Affiliations:** 1 Department of Anatomy and Cell Biology, The George Washington University School of Medicine and Health Sciences, Washington, DC, 20052 USA; 2 The George Washington University Cancer Center, The George Washington University School of Medicine and Health Sciences, Washington, DC , 20052 USA; 3 McCormick Genomics and Proteomics Center, Department of Biochemistry and Molecular Medicine, The George Washington University School of Medicine and Health Sciences, Washington, DC , 20037 USA; 4 Department of Microbiology, Immunology and Tropical Medicine, The George Washington University School of Medicine and Health Sciences, Washington, DC, 20052 USA

**Keywords:** Prostate, ERV, TRIM28

## Abstract

How endogenous retroviral elements (ERVs), a family of transposable elements, may promote tumor progression is not well understood. Tripartite motif-containing 28 (TRIM28/TIF1b/KAP1) is a key transcriptional co-repressor protein that represses ERV expression in many cell types including embryonic stem cells, neural progenitor cells, differentiated adult cells, and cancer cells. In this study, we investigated the effect of *Trim28* deletion on the expression of ERVs using an immune competent genetically engineered mouse model for prostate cancer. We found *Trim28* deletion in prostate tumors led to the expression of ERVs in prostates from both hormonally intact and castrated mice. ERVs can regulate the expression of neighboring genes, and we detected increased expression of several protein-coding genes near overexpressed ERVs. Our data suggest that *Trim28* deletion in prostate tumor epithelial cells may promote an innate immune response. However, *Trim28* deletion also led to excessive deposition of tumor extracellular matrix (ECM). Our findings suggest that ECM alterations downstream of ERV derepression could affect immune cells in the tumor microenvironment and may promote tumor progression.

**Supplementary Information:**

The online version contains supplementary material available at 10.1186/s13100-025-00382-9.

## Introduction

Endogenous retroviral elements (ERVs) are remnants of viral elements that make up 8–10% of the human and mouse genomes, and their expression is tightly regulated in differentiated cell types [1–3]. Human ERVs (HERVs) are derepressed upon oncogenic transformation of fibroblast cells, and HERV expression has been reported in multiple cancers, including prostate cancer [4, 5]. Furthermore, HERVs can act as promoters and enhancers to regulate gene expression and promote tumor progression [6, 7]. However, there remains an incomplete understanding and few *in vivo* models for studying the effects of ERV overexpression during tumor progression.

In contrast to HERV functions that promote tumor growth, activation of HERVs in tumor cells can induce anti-tumor viral-defense immune responses [8, 9]. In prostate cancers, which are immunologically “cold” with few tumor-infiltrating T cells [10], HERV activation has been suggested as an immunotherapeutic strategy for prostate cancers that have increased epigenetic H3K9 trimethylation modifications and are resistant to next-generation antiandrogens such as enzalutamide [11, 12].

The Tripartite motif-containing 28 (TRIM28/TIF1b/KAP1) is a key transcriptional co-repressor protein that represses ERV expression in many cell types including embryonic stem cells, neural progenitor cells, differentiated adult cells, and cancer cells [13–16]. Human and mouse ERV sequences are dissimilar [2], and while many ERVs are considered to be inactive due to accumulated mutations, in mice, ERVs such as intracisternal-A particle (IAP) elements are active and can cause insertional mutations [3]. Although retrotransposon activity is more limited for human HERVs, mechanisms for ERV silencing through heterochromatic gene silencing, including the recruitment of TRIM28 by KRAB Zinc finger proteins to specific DNA sequences and the deposition of H3K9 trimethylation silencing marks by SETDB1 are conserved in mammals [3, 17].

We recently developed a unique *in vivo* genetic mouse tumor model where deletion of *Trim28* in prostate epithelial cells results in tumors with decreased cellular plasticity and increased apoptosis [18, 19]. However, long-term *Trim28* deletion unexpectedly alters the tumor microenvironment and promotes tumor progression [18]. In this study, we examined how TRIM28 mediates ERV repression in this immune competent genetically engineered mouse model of prostate cancer. We found that *Trim28* deletion led to widespread expression of ERVs in prostate tumors from hormonally intact and castrated mice. Our findings suggest that ERV derepression may promote tumor progression by inducing fibrosis and remodeling the tumor extracellular matrix.

## Materials and methods

### Mouse models

All animal procedures were performed with prior approval from the Institutional Animal Care and Use Committee at the George Washington University. *Nkx3.1CreERT2/+; Pten*^*flox(f)/f*^; *p53*^*f/f*^; *R26r*^*YFP/YFP*^ (NPp53, RRID: IMSR_JAX:033755), *Nkx3.1-CreERT2/+; Pten*^*f/f*^; *p53*^*f/f*^; *Trim28*^*f/f*^; *R26r*^*YFP/YFP*^ (NPp53T), *Nkx3.1-CreERT2/+; Pten*^*f/f*^;*R26r*^*YFP/YFP*^ (NP, RRID: IMSR_JAX:033751), and *Nkx3.1-CreERT2/+; Pten*^*f/f*^; *Trim28*^*f/f*^; *R26r*^*YFP/YFP*^ (NPT) mice were bred and maintained on a C57BL/6 mixed genetic background as previously described [18]. C57BL/6 mice (RRID: IMSR_JAX:000664) were ordered from Jackson Labs and bred. Genotyping was conducted by Transnetyx or using primers as previously described [18]. Genetic background monitoring of NPp53 and NPp53T mice was conducted by Transnetyx using the Mini Mouse Universal Genotyping Array (MUGA) with over 10,000 single-nucleotide polymorphism (SNP) markers. This analysis revealed homozygosity of C57BL6 SNPs at 75.8% ± 1.1% (NPp53) and 74.2% ± 2.0% (NPp53T; *n* = 3 mice per genotype). Tamoxifen administration, castration, tissue dissection, and survival curve analysis were performed as previously described [18]. Briefly, 2-month-old mice were administered tamoxifen (200 mg/kg body weight) by oral gavage over four consecutive days to activate CreERT2-mediated gene recombination. For survival analysis following castration, mice were surgically castrated 1 month after tamoxifen treatment and euthanized when the body condition score was ≤ 2. Whole prostate tissues were weighed, and histological analyses were performed on prostate anterior lobes unless otherwise specified. Sample sizes were not predetermined, investigators were not blinded, and animals were not randomized.

### RNA sequencing analysis

Single prostates were dissected in cold 1X PBS and split into halves containing single anterior, dorsal, lateral and ventral prostate lobes. A FastPrep 24 5G bead grinder (MP Biomedicals) with stainless steel beads were used to homogenenize tissues for RNA extraction using the MagMax96 for Microarrays Total RNA Isolation kit (Applied Biosystems) following the Spin procedure. For castrated tissues, extracted RNA was treated with DNase. Total RNA samples were quantifiedusing an Agilent Bioanalyzer RNA Nano kit. Samples were prepared for sequencing by the GW Genomics Core (RRID:SCR_027546) using the Illumina Stranded Total RNA Prep, Ligation with Ribo-Zero Plus. Inputs for hormonally intact samples were normalized to 100 ng; inputs for castrated samples were normalized to 500 ng. Indexed adapters from IDT for Illumina RNA UD Indexes Set A were used for both batches. Libraries for hormonally intact samples were sequenced using an Illumina NextSeq 500, with 2 × 76 bp reads; libraries for castrated samples were sequenced using an Illumina NextSeq 2000, with 2 × 100 bp reads, at a read depth of 10–15 M paired-end reads/sample.

FASTQ files were assessed with FastQC (RRID: SCR_014583) [20]. Reads were trimmed and adapter sequences were removed using cutadapt (RRID: SCR_011841) [21] with -q 20 and –minimum-length 1. The adapter sequences were -a CTGTCTCTTATACACATCT and -A CTGTCTCTTATACACATCT. Trimmed reads were aligned to the mouse GENCODE GrCm39 primary assembly (GCA_000001635.9) using STAR (RRID: SCR_004463) [22] with annotation GTF files from GENCODE. Alignment was run with the –sjdbOverhand 100 –winAnchorMultimapNmax 100 –outFilterMultimapNmax 100 flags. Transposable element alignment was run using TEtranscripts for subfamily counts and TElocal for individual locus counts (RRID: SCR_023208) [23] with curated TE GTF files [24] with –mode multi and –stranded reverse flags. Locus-level alignment was also run using SQuIRE [25] with a custom GTF file generated from RepeatMasker including DNA, LTR, LINE, and SINE elements using the “squire Clean” command.

A matrix of TE subfamily read counts created from TEtranscripts was combined with gene counts from STAR alignment then read into R. For principal component analysis (PCA), raw reads were transformed with the rlog() command and PCA was performed with the plotPCA() command. For PCA of published data combined with the samples in our study, raw reads were normalized by variance stabilizing transformation with the vsd() command and batch effects were removed with the limma RemoveBatchEffect() function. Differential expression analysis was performed with DESeq2 (RRID: SCR_015687). Raw reads were normalized using DESeq2’s median of ratios method through the counts() command. Read counts were run through the DESeq() command and NPp53 samples were used as the reference. Hormonally intact and castrated samples, which were sequenced as separate batches, were analyzed separately.

Volcano plots were generated with VolcanoseR (RRID: SCR_025419) (Goedhart & Luijsterburg, 2020), and are shown with a significance level of *p* < 0.01 and |log2foldchange| >1.5 unless otherwise noted. Graphs were generated in R using ggplot2 and VennDiagram [26], and GraphPad Prism (RRID: SCR_002798) (GraphPad). RNA read density was visualized using the WashU Epigenome browser (RRID: SCR_006208) (Li et al., 2022).

Data from embryonic stem cells [27] and neural progenitor cells [28] were downloaded from GEO. Adapters were trimmed and reads were aligned to the mouse GENCODE GrCm39 primary assembly as described above. Raw counts from adult liver were downloaded from GEO [29].

For gene set enrichment analysis (GSEA), normalized counts from all samples were generated through DESeq2’s counts() function using the DESeq2 median of ratios method. Gene Set Enrichment Analysis was performed using the GSEA v4.4.0 Mac App (RRID: SCR_003199) and MSigDB gene sets from the Broad Institute (RRID: SCR_007073) [30, 31]. Hallmark gene sets of 50 well-defined biological processes, and a larger collection of 1309 Reactome gene sets were used. Plots were generated using ggplot2 and fgsea in R.

For analysis of co-regulated TEs and genes, RepeatMasker annotations in the UCSC and WashU Epigenome browser were used to identify LTR class elements within 200 kb of the top 20 genes derepressed in hormonally intact NPp53T tumors (ranked by adjusted p-value). TE-gene chimeric transcripts were predicted with TE Promoter Finder 2 (TEProf2) using the Kozak sequence and CPC2 algorithm [32, 33], with filtering for transcripts detected in a minimum 3 out of 5 NPp53T samples and enrichment > 1 compared to NPp53 samples.

### RT-qPCR

Anterior prostate lobes were dissected into cold 1X PBS, split along the anterior-posterior axis, and flash frozen. A FastPrep 24 5G bead grinder (MP Biomedicals) with stainless steel beads was used to homogenenize tissue. RNA was extracted using the MagMax96 for Microarrays Total RNA Isolation kit (Applied Biosystems) following the No Spin procedure. All samples were treated with DNAseI. RNA input was standardized to 400 ng and cDNA was generated using the SuperScript IV First-Strand Synthesis System with random hexamer primers. cDNA samples were diluted 1:10. Real-time quantitative PCR (RT-qPCR) was performed using the PowerUp SYBR Green Master Mix (Applied Biosystems) in the BioRad CFX96 instrument. Gene expression values were obtained using the delta delta CT method using *Gapdh* expression as the reference gene. Primer sequences for TE loci were designed using PRIMER-BLAST (NCBI) and Primer3 [34] and are provided in Table S1.

### Immunostaining analysis

Immunohistochemistry and immunofluorescence analysis were performed as previously described (Yende et al., 2023). Antibodies and their concentrations are in Table S2. Confocal images were captured on a Zeiss LSM 980 and analyzed using ImageJ Fiji (RRID: SCR_002285) and Adobe Photoshop. Picrosirius red staining was performed on 5 µM paraffin sections using the Picrosirius Red Stain Kit (Polysciences). Slides were imaged using a Leica DMi8 microscope.

For quantitation of dsRNA staining intensity, tissues were scored as having low (< 1% of epithelial cells), intermediate, or high staining (staining in $\:>\:$50% of epithelial cells). Wildtype C57BL/6J mouse prostate tissue from adult males aged 2–3 months and no-primary antibody controls were used as negative controls. For quantification of CD3 and CD206 positive (+) cells, YFP+ DAPI+ regions of prostate ducts were traced in Photoshop using the Quick Selection tool. Ductal area was measured using the ImageJ Measure tool. Cell quantitation was performed manually using the ImageJ Cell Counter tool.

### Dot blot for dsRNA

RNA was extracted from frozen anterior prostate fragments by homogenization in TRI Reagent using a FastPrep 24 5G bead grinder (MP Biomedicals) with stainless steel beads. 1-bromo-3-chloropropane was added, incubated for 15 min at room temperature, and centrifuged at 12,000 g for 15 min at 4 °C to ensure phase separation. The aqueous layer was removed and RNA was precipitated with isopropanol at 12,000 g for 10 min at 4 °C. RNA pellets were washed with 100% ethanol, centrifuged at 7500 g for 5 min at 4 °C, dried and resuspended in nuclease-free water, heating for 3–5 min at 55 °C to ensure resuspension. RNA was diluted to 15ng/µL with nuclease-free water. A Nylon Hybond + membrane was rinsed with water and loaded with 5 and 1 µg of RNA from each sample using a Biorad Bio-Dot Apparatus. RNA was fixed to the membrane by UV crosslinking. The membrane was blocked for 1 h at room temperature in 5% milk (w/v, in 0.1% PBS-Tween-20), incubated overnight with dsRNA J2 antibody at 1:500 concentration (Table S2), and incubated with anti-mouse HRP conjugated secondary antibody, washing with 0.1% PBS-Tween-20. Signal was detected via chemiluminescence using SuperSignal West Femto Maximum Sensitivity Substrate (Thermo Fisher). The membrane was then incubated for 15 min with 0.02% methylene blue (w/v), rinsed 4–6 times with water, and imaged to visualize total RNA loading. Dot blot staining intensity was quantified using the integrated density function in ImageJ.

### Statistical analysis

Statistical analyses were performed using Prism 9 (GraphPad, RRID: SCR_002798) using a two-tailed unpaired Student’s t test unless otherwise noted. Data were assumed to be normally distributed with similar variance. Sample sizes are noted in figure legends.

## Results

### TRIM28 inhibition induces the expression of endogenous retroviral elements in the NPp53 mouse model of prostate cancer

**Fig. 1:**
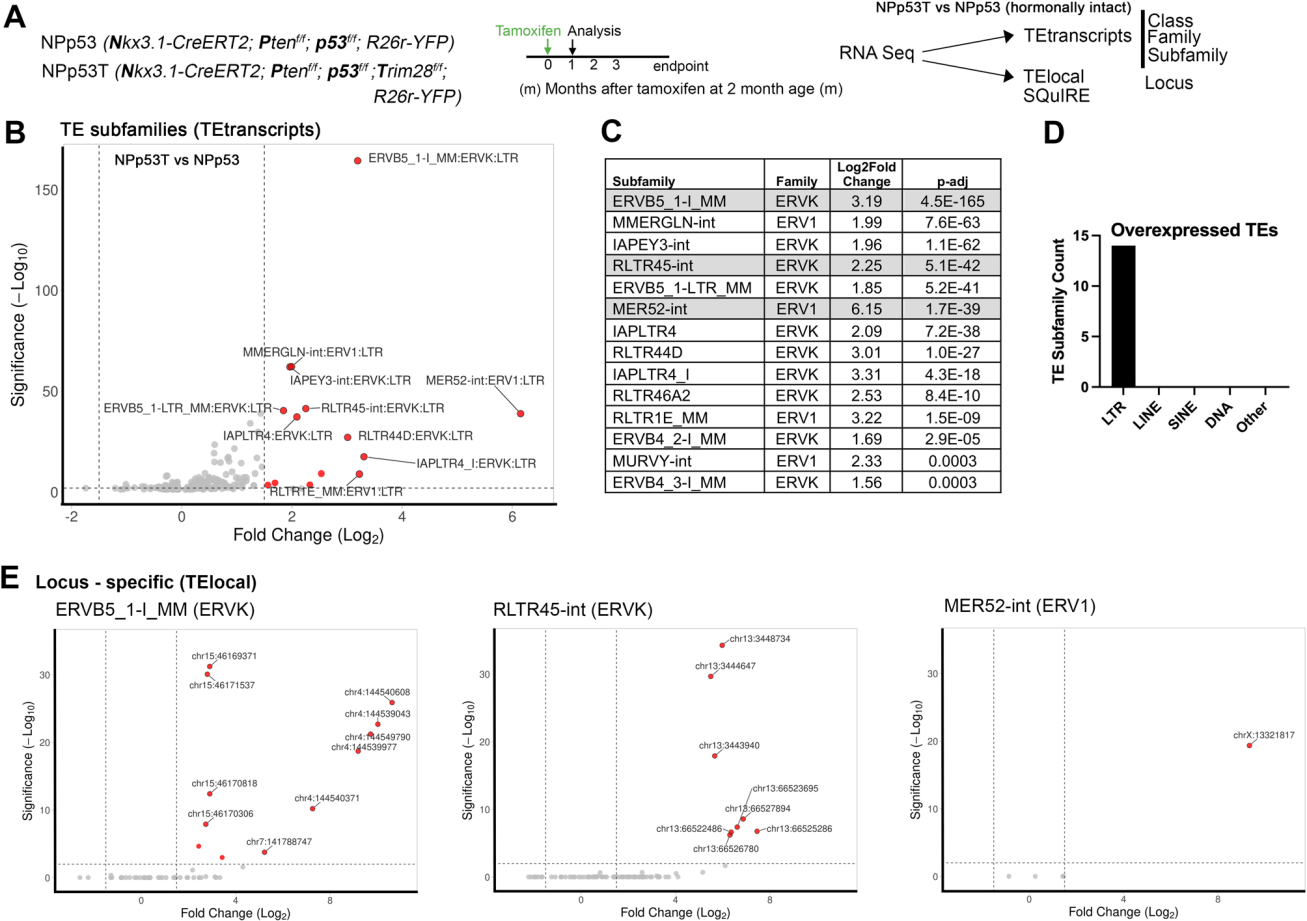
*Trim28* deletion induces expression of endogenous retroviral elements in the NPp53 mouse model of prostate cancer.** A** Experimental outline. **B** Volcano plot of differentially expressed transposable element (TE) subfamilies in NPp53T compared to NPp53 tumor prostates. Dotted lines indicate thresholds at log2 fold change ≥1.5 and p<0.01. n=5 prostates for each genotype. Red, significantly upregulated in NPp53T prostates. **C** Significantly overexpressed TE subfamilies after *Trim28* deletion. The subfamilies shown in panel E are highlighted. **D** Significantly overexpressed TE subfamilies by class. **E **Volcano plots of individual loci from C. See also Fig. S1.

Inactivation of both *PTEN* and *TRP53* tumor suppressor proteins is frequently observed in advanced prostate cancers [36]. The NPp53 (*Nkx3.1-CreERT2/+; Pten*^*f/f*^; *p53*^*f/f*^) genetically engineered mouse model with combined inactivation of *Pten* and *p53* can be used to model human castration-resistant prostate cancer [35]. To determine the effect of *Trim28* deletion on ERV expression, we used the NPp53T (*Nkx3.1-CreERT2/+; Pten*^*f/f*^; *p53*^*f/f*^; *Trim28*^*f/f*^) mouse model of prostate cancer with deletion of *Pten*,* p53* and *Trim28* (Fig. 1A [18]),. We compared *Trim28* deleted NPp53T prostates to control NPp53 (*Nkx3.1-CreERT2/+; Pten*^*f/f*^; *p53*^*f/f*^) prostates [35] at 1 month after inducing tumors, when NPp53 and NPp53T prostates were similar in size [18]. Our analysis of bulk RNA sequencing transcripts revealed *Trim28* deletion altered transposable element expression (Fig. 1B, *n* = 5 mice per genotype).

The expression of 14 transposable element subfamilies was increased in *Trim28* deleted NPp53T prostates compared to NPp53 prostates (Fig. 1B-C, log2 fold change ≥ 1.5, *p* < 0.01). All 14 of these transposable element subfamilies were long terminal repeat (LTR) elements, belonging to the ERVK and ERV1 families (Fig. 1C, D). While some subfamilies such as MER52-int were highly overexpressed in *Trim28* deleted NPp53T prostates with a log2 fold change over 6, a lower level of overexpression was detected in many of the overexpressed subfamilies (Fig. 1B-C). The expression of other classes of transposable elements such as LINE, SINE, and DNA elements was not affected by *Trim28* deletion (Fig. 1D). We also did not detect decreased expression of LTR element subfamilies with *Trim28* deletion (log2 fold change ≥ 1.5, *p* < 0.01, Supplementary file 1). These findings indicate that TRIM28 represses ERVs in NPp53 prostate tumor cells, with *Trim28* deletion resulting in expression of ERV subfamilies.

To further understand how TRIM28 represses ERVK and ERV1 expression in the prostate and the consequences of ERV expression, we assessed locus-specific expression information for interspersed TE loci using TElocal [23] and SQuIRE [25] (Fig. S1A, B). For many of the overexpressed ERV subfamilies including ERVB5_1-I_MM and RLTR45-int, we detected increased expression from distinct loci across several chromosomes in *Trim28* deleted NPp53T prostates (Fig. 1E, Fig. S1A-C). However, for ERV subfamilies MER52-int, RLTR44D, and MURVY-int, we detected increased expression from only a single locus upon *Trim28* deletion (Fig. 1E, Fig. S1A, B). Overall, these findings suggest that *Trim28* deletion in NPp53T prostate tumors results in the derepression of ERVs across numerous genomic loci.

### TRIM28-mediated repression of ERVs is androgen-independent 

**Fig. 2:**
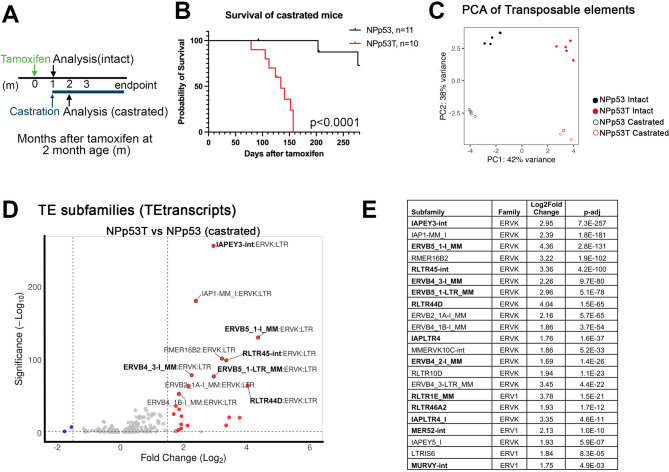
TRIM28-mediated repression of ERVs is androgen-independent. **A** Experimental outline. **B** Kaplan-Meier survival curve of castrated NPp53 and NPp53T mice. P-value for the difference in probability of survival was calculated by log-rank test. **C** Principal components analysis (PCA) of scaled transposable element (TE) subfamily expression obtained from bulk RNA sequencing. n=4 or 5 prostates for each genotype and condition. **D** Volcano plot of differentially expressed TE subfamilies in prostate tumors from castrated NPp53T mice compared to castrated NPp53 mice. Dotted lines indicate thresholds at log2 fold change ≥1.5 and p<0.01. Red, significantly upregulated in NPp53T prostates; blue, significantly downregulated in NPp53T prostates. **E** Significantly overexpressed TE subfamilies after Trim28 deletion in prostate tumors from castrated mice. Subfamilies in bold were also overexpressed in prostates from hormonally intact NPp53T mice

Given the importance of androgen deprivation therapy in the treatment of advanced prostate cancers, we investigated the effect of combining *Trim28* deletion and androgen deprivation on ERV expression. When mice bearing NPp53T tumors with *Trim28* deletion were castrated, the tumors developed castration-resistance, resulting in a median survival of 134 days after tumor induction, which was significantly shorter than the survival for castrated mice with NPp53 tumors (Fig. 2A, B), and similar to our previously reported median survival of 122 days for hormonally intact NPp53T tumors [18]. Like hormonally intact NPp53T mice, castrated NPp53T mice had distended bladders filled with urine at advanced stages, suggestive of death due to prostate overgrowth and bladder obstruction (*n* = 6 castrated NPp53T mice [18]).

Since treatment of LNCaP prostate cancer cells with the antiandrogen enzalutamide promotes the expression of transposable elements [11], we hypothesized that combining *Trim28* deletion with androgen deprivation (castration) would increase ERV expression. We collected prostate tissues from castrated NPp53 and NPp53T prostates 1 month after castration, when prostate weights of NPp53 and NPp53T prostates were similar (Fig. 2A [18]),. We analyzed transposable element expression from bulk RNA sequencing transcripts using principal components analysis, which revealed changes in transposable element expression caused by TRIM28 deletion, as well as changes resulting from androgen deprivation (Fig. 2C).

In pairwise comparisons of prostates from castrated NPp53T mice with *Trim28* deletion to castrated NPp53 mice, the expression of 22 transposable element subfamilies was increased in prostates from castrated NPp53T mice (Fig. 2D, log2 fold change ≥ 1.5, *p* < 0.01). Similar to our findings in hormonally intact NPp53T mice, all 22 of the transposable element subfamilies with significantly increased expression upon *Trim28* deletion were long terminal repeat (LTR) elements, belonging to the ERVK and ERV1 families (Figs. 1 and 2E). Of the 22 transposable element subfamilies, 13 were significantly increased with TRIM28 deletion in both hormonally intact and castrated NPp53T mice (log2 fold change ≥ 1.5, *p* < 0.01; bolded in Fig. 2E), and an additional 7 had increased expression with a less stringent threshold of log2 fold change ≥ 0.9, *p* < 0.05. (Supplementary file 1). These findings indicate a high overlap of TRIM28 regulated ERVs in prostate tumors from hormonally intact and castrated mice.

We further compared TRIM28 repression of ERVs in NPp53 prostate tumors to embryonic stem cells and neuronal progenitor cells [27, 28](Fig. S1D). Of the 22 ERV subfamilies with significantly increased expression in prostates from castrated NPp53T mice, 12 were derepressed after *Trim28* deletion in embryonic stem cells or neuronal progenitor cells (log2 fold change > 1.0, *p* < 0.05; Fig. S1B), suggesting conserved mechanisms for ERV repression.

### *Trim28* deletion promotes dsRNA formation

**Fig. 3:**
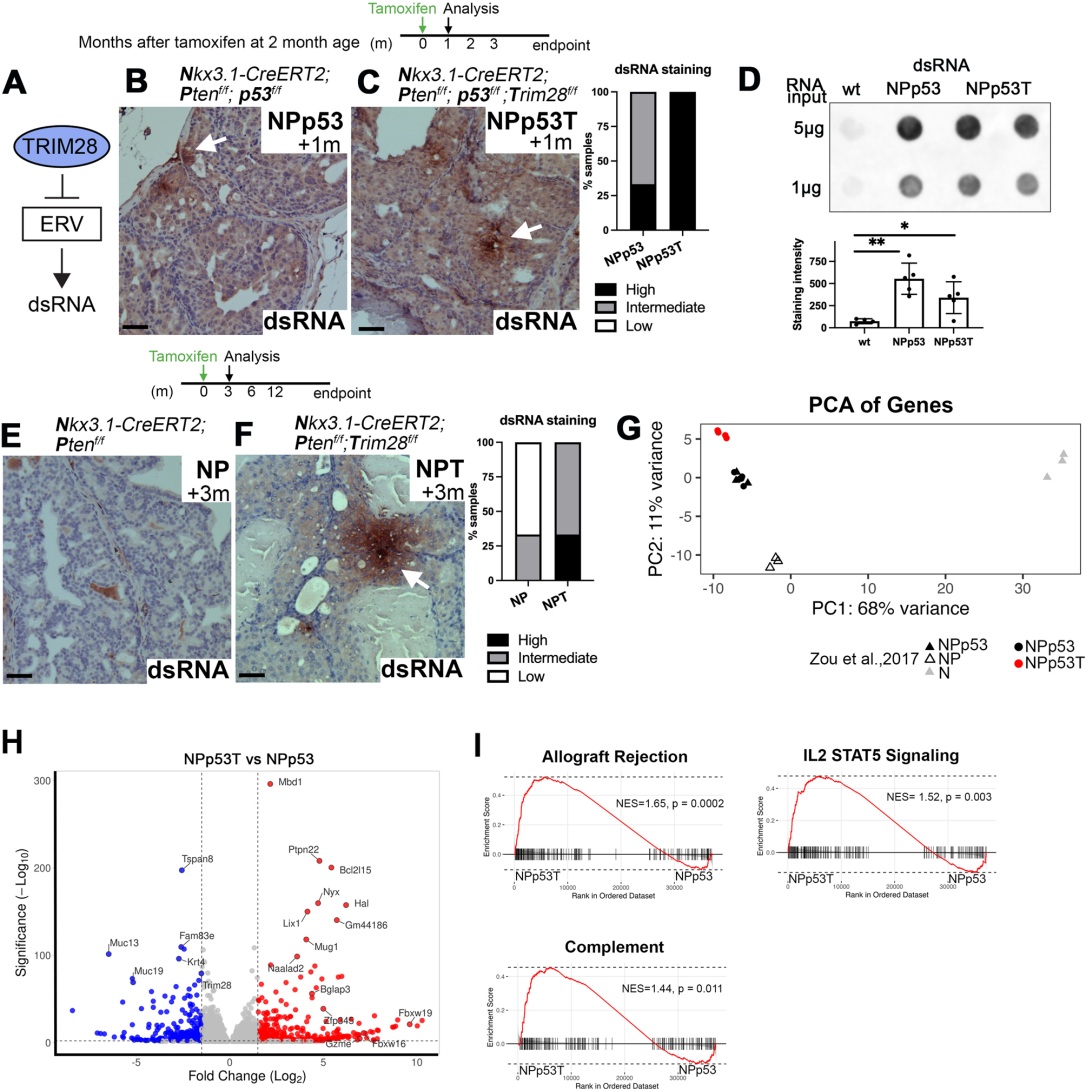
*p*53 and *Trim28* deletion promote dsRNA formation. **A** Diagram of TRIM28-mediated repression of endogenous retroviral elements (ERVs) and dsRNA formation. **B**, **C**, **E**, **F** Experimental timeline, immunohistochemistry staining for dsRNA and quantitation of staining intensity. Arrows point to regions of dsRNA staining. Representative images from anterior prostates from hormonally intact (B) NPp53 (n=6), (C) NPp53T (n=6), (E) NP (n=6), and (F) NPT (n=3) mice are shown. Nuclei were stained with hematoxylin. Scale bars represent 50 μm. **D** Representative image of dsRNA dot blot and quantification of staining intensity. n=4-5 prostates per genotype. Error bars represent s.d.; *P* values were calculated using a one-way ANOVA test. **p < 0.01; *p < 0.05. **G** Principal components analysis (PCA) of scaled gene expression obtained from bulk RNA sequencing using samples from this study and Zou et al., 2017. **H** Volcano plot of differentially expressed genes in prostate tumors from NPp53T mice compared to NPp53 mice. Dotted lines indicate thresholds at log2 fold change ≥1.5 and p<0.01. Red, significantly upregulated in NPp53T prostates; blue, significantly downregulated in NPp53T prostates. **I** Gene Set Enrichment Analysis (GSEA) of bulk RNA sequencing data from NPp53T (n=5) versus NPp53 (n=5) mice with selected Hallmark gene sets. See also Fig. S2 and Fig. S3

To evaluate if overexpression of ERVs leads to increased double-stranded (ds)RNA formation and can activate an interferon response that mimics a viral infection [8, 37], we stained prostate tissues using the J2 monoclonal antibody that detects dsRNA. At 1 month after tumor induction, we observed elevated dsRNA staining in both NPp53 and *Trim28* deleted NPp53T tumors (Fig. 3A-D). The dsRNA staining intensity was high (> 50% of epithelial cells) in all *Trim28*-deleted NPp53T tumors, which contained foci with high signal intensity at 1 month after tumor induction, whereas the intensity was high or intermediate (1–50% of epithelial cells) in NPp53 tumors (Fig. 3B, C, arrows). Such dsRNA staining was not detected (low, < 1% of epithelial cells) in wild type C57BL/6 prostates (Fig. 3D, Fig. S2A). dsRNA staining was also detected in later-stage NPp53T and age-matched NPp53 tumors at 3 months after tumor induction (Fig. S2B, C). Evaluation of ERV expression at specific loci using RT-qPCR revealed that ERVs remain overexpressed 3 months after *Trim28* deletion (Fig. 1, Fig. S2D).

Given our finding that dsRNA staining was elevated in both NPp53T and NPp53 tumors, we assessed the effect of *p53* deletion on dsRNA formation by comparing *Nkx3.1-CreERT2*; *Pten*^f/f^ (NP) tumors with wildtype p53 to *Nkx3.1-CreERT2*; *Pten*^f/f^; *Trim28*^f/f^ (NPT) tumors [18]. Due to the slower growth of NP tumors [38], we first analyzed early lesions at 3 months after induction. We found that in NP tumors, *Trim28* deletion was sufficient to induce dsRNA formation in early lesions (Fig. 3E, F) and at later timepoints (Fig. S2E, F). However, similar to our observations with *Trim28* deletion in NPp53 prostates (Fig. 2A, B [18]), NPT mice had a shorter survival compared to NP mice (Fig. S2G).

We also assessed the effect of *p53* deletion on transposable element expression in tumors by evaluating transposable element expression in NPp53, NP prostate tumors and control *Nkx3.1-CreERT2* (N) prostate tissues [35] (Fig. S2H). The *Nkx3.1*-*CreERT2* allele generates a null allele, and prostates from *Nkx3.1*^+/-^ mice can display hyperplasia, but the lesions do not develop into invasive carcinomas [39, 40]. Whereas *Trim28* deletion in NPp53 prostate tumors primarily affected ERV elements (Fig. 1D), comparison of NPp53 to NP prostate tumors revealed differential expression of many transposable element subfamilies from classes including long interspersed nuclear elements (LINEs), short interspersed nuclear elements (SINEs), and DNA elements (Fig. S2I-K). Fewer differences were observed when NP tumors were compared to N prostates (Fig. S2L-M). Altogether, these findings suggest that both *p53* deletion and *Trim28* deletion promote dsRNA formation.

To assess how ERV expression and dsRNA formation may contribute to the phenotypes observed in *Trim28* deleted tumors, we analyzed gene expression in prostates from bulk RNA sequencing samples (Fig. 1A). Principal components analysis of gene expression revealed clustering of samples by genotype (Fig. 3G), and by castration status of the mice (Fig. S3A). We focused our analysis on the comparison of NPp53T prostate tumors to NPp53 prostate tumors in hormonally intact mice and conducted differential gene expression analysis, which revealed upregulation of 375 genes and downregulation of 252 genes (log2 fold change ≥ 1.5, *p* < 0.01; Fig. 3H, Supplementary file 2). Genes overexpressed in NPp53T prostate tumors included *Bglap3* and *Ptpn22*, which we previously identified as overexpressed in tumor cells upon *Trim28* deletion [18], and granzyme E (*Gzme*), expressed in cytotoxic T cells (Fig. 3H) [41]. We did not detect increased expression of dsRNA sensors or downstream interferon-stimulated genes in NPp53T prostates compared to NPp53 prostates (Fig. S3B). However, Gene Set Enrichment Analysis (GSEA) [30] comparing *Trim28* deleted NPp53T tumors to NPp53 tumors revealed a positive enrichment for Hallmark gene sets including allograft rejection, IL2 STAT5 signaling and immune complement signatures (Fig. 3I, Fig. S3C, D), suggesting an innate immune response in NPp53T tumors.

### ERV promoters may regulate gene expression in NPp53T tumors 

**Fig. 4:**
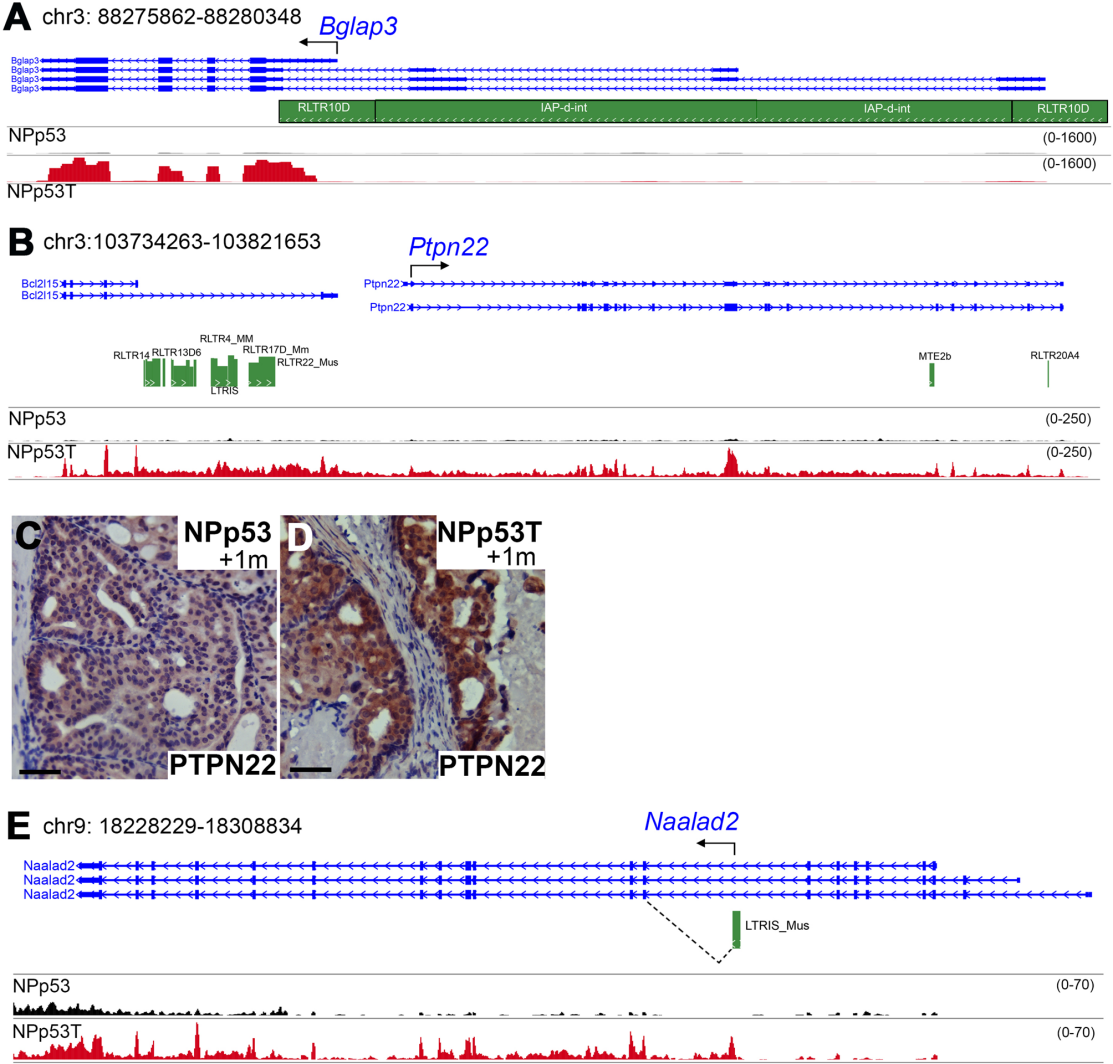
ERVs may promote gene expression in NPp53T tumors. **A**, **B**, **E** Representative tracks from bulk RNA sequencing of NPp53 (black) and NPp53T (red) prostate tumors (n=5 prostates for each genotype). RefSeq transcripts (blue) and RepeatMasker LTR repeats on the same strand (green) are shown. A predicted TE gene fusion transcript is shown in E. **C**, **D** Immunohistochemistry staining for PTPN22. Representative images from n=5 prostates per genotype are shown. Nuclei were stained with hematoxylin. Scale bars indicate 50 μm. See also Fig. S4

In tumors and in normal cells, ERVs can be co-regulated with and may promote the expression of nearby genes [6, 7, 27–29, 42]. To identify ERVs expressed in NPp53T tumors that may function as promoters for nearby genes, we examined ERVs within 200 kilobases (kb) of several overexpressed genes (Fig. 4). The 3’LTR of the RLTR10D::IAP-d-int ERV element in the *Bglap3* gene is known to function as a promoter to regulate *Bglap3* expression in several differentiated cell types [29]. We previously found increased BGLAP3 expression after *Trim28* deletion in NPp53T mouse prostates [18], and thus, we examined RLTR10D::IAP-d-int expression in NPp53T mouse prostates. We found that *Trim28* deletion in NPp53T mouse prostates resulted in expression of RLTR10D, which may promote expression of the short isoform of *Bglap3* (Fig. 4A).

We previously also reported increased gene expression of *Ptpn22* in prostate tumor epithelial cells after *Trim28* deletion [18], although *Ptpn22* expression is not altered after *Trim28* deletion in embryonic stem cells, neuronal progenitor cells, or adult liver (Supplementary file 3; [27–29]). Examination of upstream sequences revealed expression of several ERVs and the adjacent gene *Bcl2l15*, suggesting transcriptional coregulation (Fig. 4B). We further confirmed increased PTPN22 protein expression in NPp53T mouse prostate tumors compared to NPp53 tumors by immunostaining (Fig. 4C, D). We identified increased expression of ERVs upstream of other genes overexpressed in NPp53T prostate tumors including *Nyx*,* Fbxw19 and Mdb1* (Fig. 3G, Fig. S4). Additionally, the TE Promoter Finder 2 (TEProf2) pipeline, which predicts TE gene fusion transcripts [32], predicted splicing of LTRIS_Mus and exon 7 of *Naalad2* resulting in expression of a transcript predicted to encode a truncated protein in NPp53T prostate tumors (Fig. 4E). Altogether, our analysis revealed that the expression of ERVs in tumors may promote the expression of nearby genes.

### *Trim28* deletion in epithelial cells affects the tumor extracellular matrix 

**Fig. 5:**
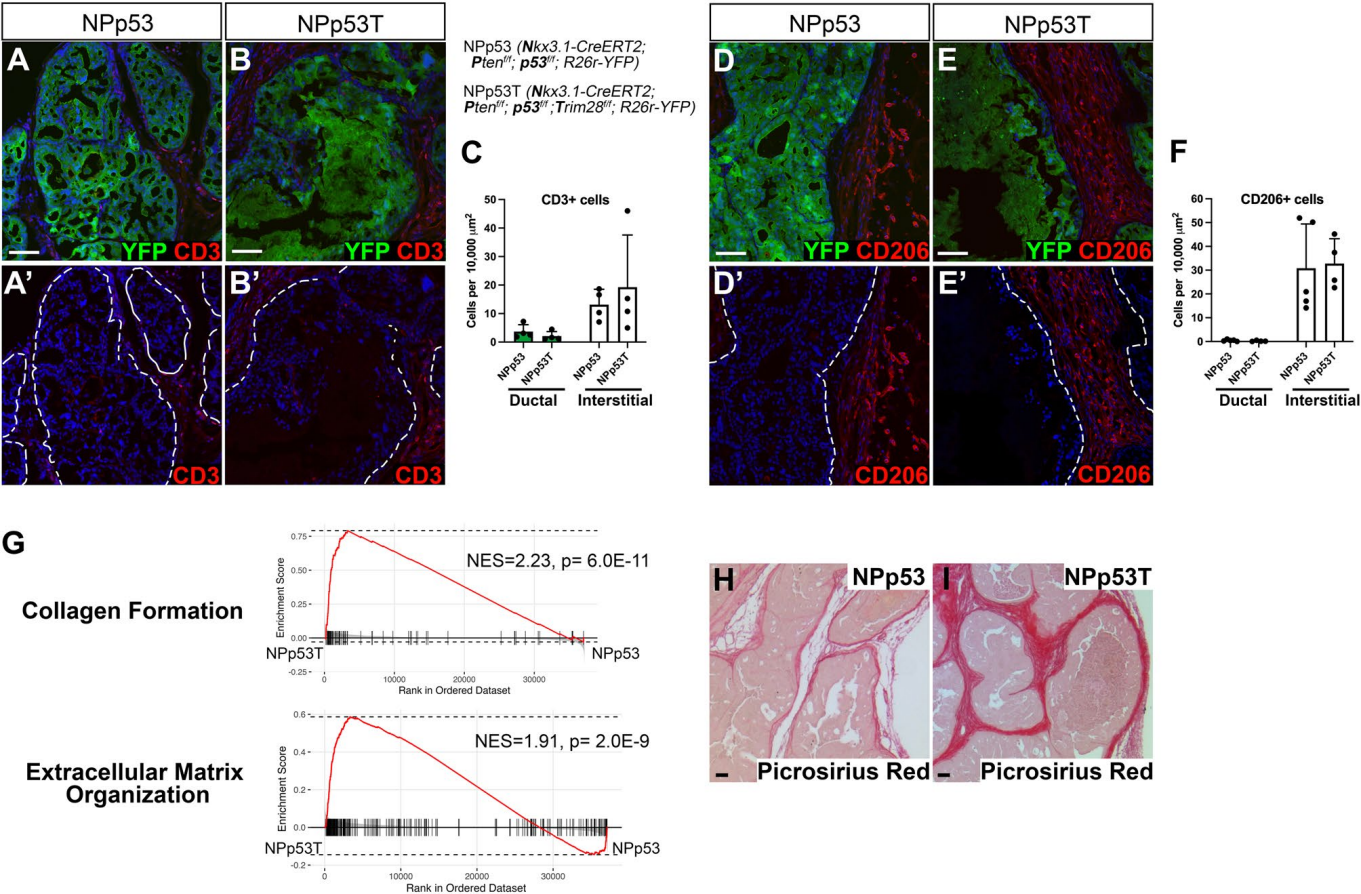
*Trim28* deletion in epithelial cells affects the tumor extracellular matrix. **A**, **B**, **D**, **E** Immunofluorescence staining of anterior prostate tumors from NPp53 (**A**, **D)** and NPp53T (**B**, **E)** mice 1 month after tumor induction for (**A**, **B)** YFP lineage-labeled epithelial cells, T cell marker CD3, DAPI, and (**D**, **E)** YFP lineage-labeled epithelial cells, macrophage marker CD206, and DAPI. **A**’-**E**’ Images from A-E shown without YFP. Dashed white lines indicate the basement membrane of the prostate duct. n≥4 mice per genotype. **C**, **F** Quantitation of C CD3+ cells shown in (**A**, **B**) and (**F**) CD206+ cells shown in (**D**, **E**). n= 4-5 mice, >600 CD3+ cells and >750 CD206+ cells for each genotype. Error bars represent s.d. **G** Gene Set Enrichment Analysis (GSEA) of bulk RNA sequencing data from NPp53T (n=5) versus NPp53 (n=5) mice with selected Reactome gene sets. **H**, **I** Picrosirius red staining for collagen fibers in NPp53 and NPp53T anterior prostates 1 month after tumor induction (n=4 prostates for each genotype). Scale bars indicate 50 μm. See also Fig. S5

Transposable element expression is correlated with an increased immune response in several cancers, including prostate cancer [43]. To assess whether *Trim28* deletion and subsequent expression of ERVs or misregulated protein-coding genes could recruit immune cells to the tumor, we conducted immunostaining analysis for CD3 + T cells. We focused on the initial response to *Trim28* deletion at 1 month after tumor induction, as YFP-lineage marked tumor cells in late-stage NPp53T tumors display sarcomatoid features, with malignant cells invading the stroma [18]. At 1 month after tumor induction, we found that the density of CD3 + cells in lineage-labeled YFP + tumor regions and in the tumor microenvironment did not differ between NPp53T and control NPp53 tumors (Fig. 5A-C). Additionally, immunostaining analysis for CD206 + macrophages revealed that most CD206 + macrophages were localized outside of YFP + ducts (Fig. 5D-F). These findings suggest that *Trim28* deletion and associated ERV expression is not sufficient to increase CD3 + T cell infiltration into early stage NPp53 prostate tumors.

Strikingly, gene set enrichment analysis (GSEA) comparing NPp53 tumors with and without *Trim28* deletion against 1309 Reactome pathways identified several pathways involved in collagen formation and extracellular matrix organization with high Normalized Enrichment Scores (NES; Fig. 5G, Fig. S5A). Using Picrosirius Red staining, we visualized collagen fibers 1 month after tumor induction, which confirmed the increase in interstitial collagen fibers in *Trim28* deleted NPp53T compared to NPp53 prostates (Fig. 5H, I). This increase was even more pronounced in late-stage NPp53T tumors 3 months after tumor induction (Fig. S5C-E). These findings are consistent with our previous observations of increased collagen expression in NPp53T prostates [18]. Similar to derepression of ERVs causing fibrosis in the kidney and lung [44, 45], our findings suggest that derepression of ERVs may promote tumor progression in NPp53T prostates by remodeling the extracellular matrix.

## Discussion

Our study of ERV expression in an immune competent genetically engineered mouse model of prostate cancer revealed that *Trim28* deletion in NPp53 prostate tumors in both hormonally intact and castrated mice resulted in increased expression of ERVs at numerous loci. This increased expression was specific to ERVs without affecting other classes of repetitive elements such as SINEs and LINEs. ERV expression was associated with formation of dsRNA, expression of proteins including *Bglap3* and *Ptpn22* not typically expressed in prostate epithelial cells, and formation of collagen and remodeling of the extracellular matrix.

While TRIM28 repression of ERVs has been examined in several cell types including ES cells and neural progenitor cells, our study examined ERV expression in a tumor context. Our analysis highlighted context-specific differences in TRIM28 repression of ERVs, with *Trim28* deleted NPp53T prostates expressing the short isoform of *Bglap3*, similar to in the adult liver, whereas the long transcript of *Bglap3* is derepressed upon *Trim28* deletion in neuronal progenitor cells and embryonic stem cells [27–29]. We also identified *Ptpn22* as a gene repressed by TRIM28 in NPp53 prostate tumors, but not in ES cells, neural progenitor cells, or liver.

Although our analysis in this study focused on the expression of ERVs at 1 month after *Trim28* deletion in NPp53T prostate tumors, RT-qPCR analysis confirmed persistent ERV expression up to 3 months post-*Trim28* deletion. We also found that *Trim28* deletion in NPp53 prostates resulted in the expression of similar subfamilies of ERVs in hormonally intact and castrated mice (Fig. 2), suggesting that TRIM28-mediated repression of ERVs occurs independently of androgens in NPp53 prostates. However, our study did not examine histone marks near ERV loci, and it is possible that some of the loci we identified were upregulated along with nearby genes in response to *Trim28* deletion. Our study also did not examine whether ERV proteins are synthesized from these ERV transcripts.

Despite increased ERV expression, detection of dsRNA, and expression of several genes involved in the innate immune response in *Trim28* deleted NPp53T prostates, our RNA sequencing analysis did not reveal a viral mimicry response with activation of dsRNA sensors. This could be due to the timing of tissue analysis, at 1 month following tumor induction, or that our comparison to NPp53 prostates, where *p53* deletion promotes repetitive element expression and dsRNA formation, affected our ability to detect elevated dsRNA signaling. In ovarian cancers, *p53* deletion promotes chronic viral mimicry and tolerance [46]. Additionally, *TRIM28* deletion alone has been shown to have a limited effect in increasing dsRNA formation in cancer cell lines, though when combined with radiation, dsRNA formation is enhanced [15].

**Fig. 6:**
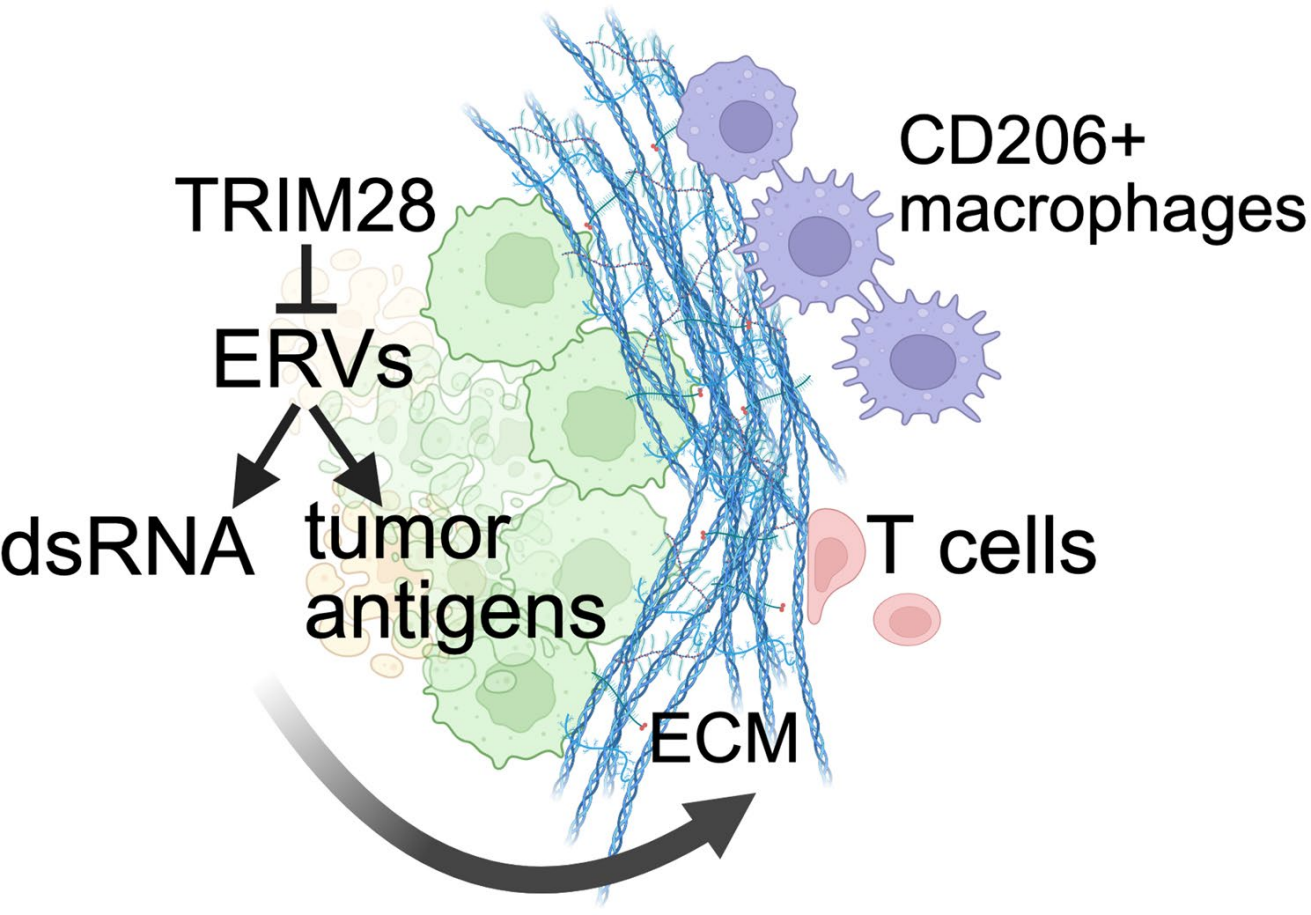
Model for effects of ERV derepression and extracellular matrix deposition on immune cells. Created in BioRender. Shibata, M. (2025) https://BioRender.com/1fm414d

Our study suggests that ERV expression and chronic inflammation may promote fibrosis and changes to the extracellular matrix in tumors, as observed in kidney and lung disease [44, 45]. Higher collagen density in the tumor extracellular matrix can promote immunosuppressive activity of macrophages and suppress T cells migration [47, 48]. In the prostate, macrophages are abundant during development [49] and can support tumorigenesis [50–52]. We previously reported an increase in CD206 expressing M2-like macrophages in NPp53T prostates at 3 months, but not at 1 month after tumor induction [18]. Thus, our findings of increased collagen formation and extracellular matrix remodeling at 1 month after tumor induction indicate that these tumor extracellular matrix changes precede the increase in CD206-expressing macrophages, and likely enhance tumor progression by preventing immune cell infiltration and promoting immune suppression (Fig. 6). It is also possible that genes other than ERVs affected by *Trim28* deletion could affect extracellular matrix remodeling. Nevertheless, these findings have wider implications for understanding the association between ERV expression, chronic inflammation, and cancer.

## Supplementary Information

Supplementary file 1

Supplementary file 2

Supplementary file 3

Supplementary figures and tables

## Data Availability

The RNA sequencing datasets generated were deposited into the Gene Expression Omnibus (GEO) data repository user accession number GSE298402. Other published datasets analyzed are available in the GEO repository under accession numbers GSE41903 (embryonic stem cells [27]), GSE45930 (neural progenitor cells [28]),, GSE74278 (adult liver [29]), and GSE92721 (prostate tumors [35]).
