# Supplementary material for: Expression of endogenous retroviral elements is associated with extracellular matrix remodeling in prostate cancer
Source: Mob DNA. 2026 Jan 8;17:1. doi: 10.1186/s13100-025-00382-9 (PMC12781564; doi:10.1186/s13100-025-00382-9)
Supplement: Supplementary file 4 — Supplementary figures and tables [file 13100_2025_382_MOESM4_ESM.pdf]

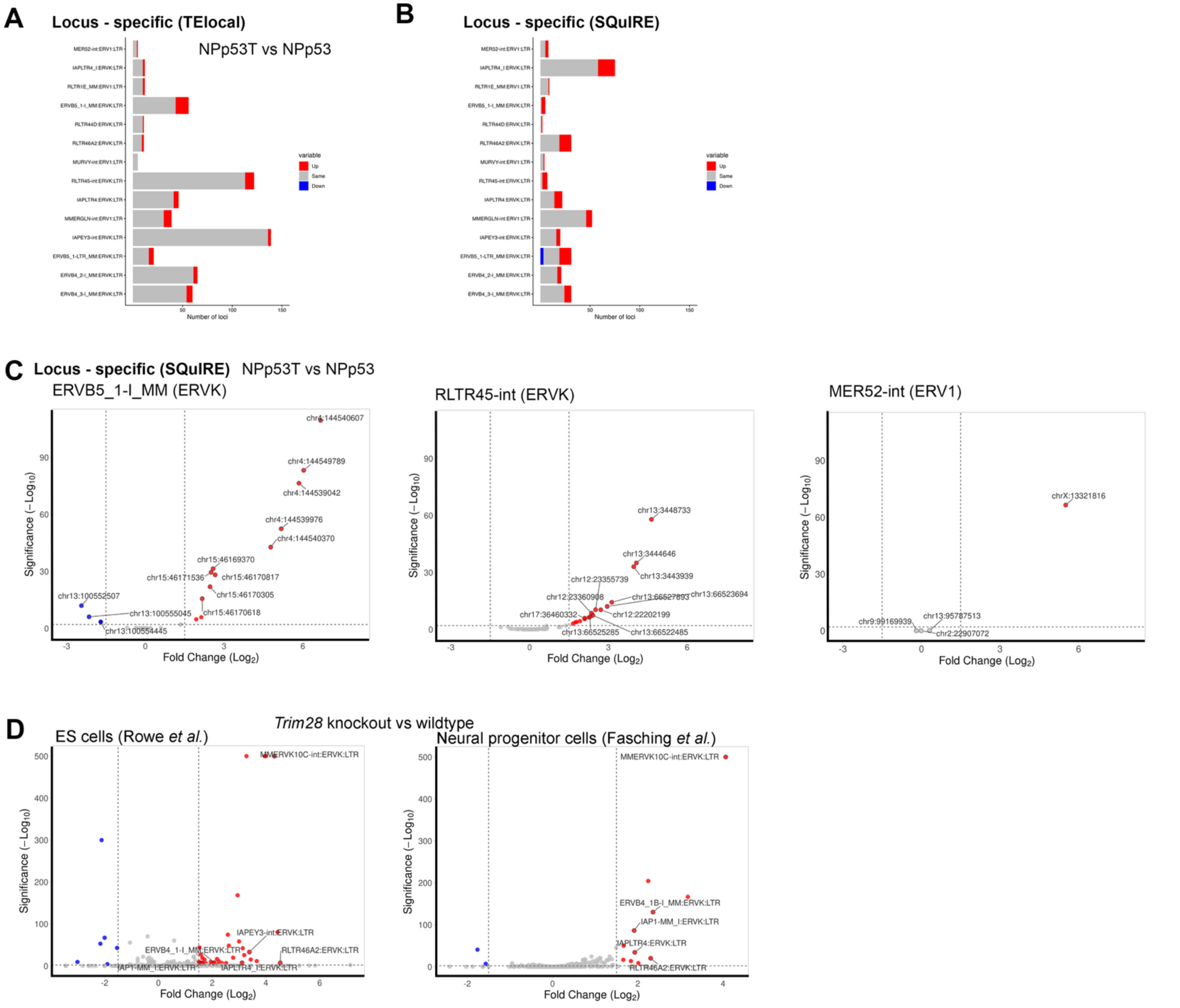

Figure S1. ***Trim28* deletion promotes transcriptional changes in prostate tumors. (A,B)** Differential expression of distinct loci from upregulated and downregulated subfamilies ($p<0.01$, log2 fold change ≥1.5). **(C)** Volcano plots of selected individual loci. **(D)** Volcano plots comparing transposable element (TE) subfamily expression in *Trim28* knockout to wildtype embryonic stem (ES) cells (Rowe *et al.,* 2013) and neural progenitor cells (Fasching *et al.,* 2015). ERV subfamilies overexpressed in NPp53T tumors are labeled. Dotted lines indicate thresholds at log2 fold change ≥1.5 and $p<0.01$. Red, significantly upregulated in *Trim28* knockout; blue, significantly downregulated in *Trim28* knockout. Related to Figure 1.

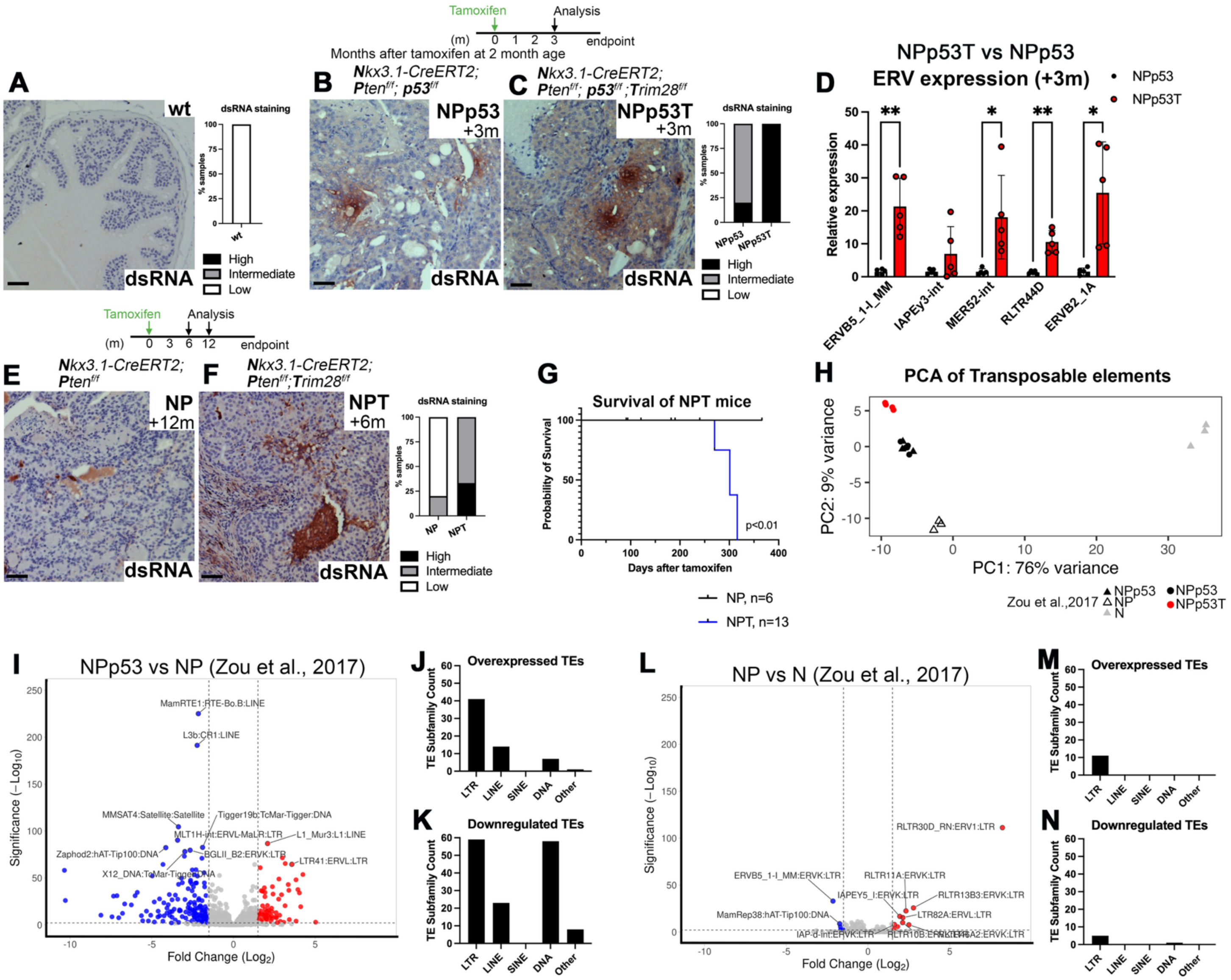

Figure S2. ERV expression after *p53* and *Trim28* deletion persists during tumor progression. (A-C, E-F) Experimental timeline, immunohistochemistry staining for dsRNA and quantitation of staining intensity. Representative images from anterior prostates from adult wildtype (wt; n=4) NPp53 (3 months after tumor induction; n=5), NPp53T (3 months after tumor induction; n=6), NP (12 months after tumor induction; n=5), and NPT (6-9 months after tumor induction; n=5) mice are shown. Nuclei were stained with hematoxylin. Scale bars represent 50 μm. **(D)** RT-qPCR analysis of ERV expression in NPp53 and NPp53T anterior prostate tissues at 3 months after tumor induction. Assays were performed on n=5 prostates for each genotype with three technical replicates, using *Gapdh* as the reference gene. **p<0.01, *p<0.05. Error bars represent *s.d.* **(G)** Kaplan-Meier survival analysis of NP and NPT mice. P-value for the difference in probability of survival was calculated by log-rank test. **(H)** Principal components analysis (PCA) of scaled transposable element expression obtained from bulk RNA sequencing using samples from this study and Zou *et al.,* 2017. **(I-N)** Volcano plots comparing expression of TE subfamilies using bulk RNA expression data (Zou *et al.,* 2017). (I-K) NPp53 prostate tumors with p53 deletion compared to NP prostate tumors. Red, significantly upregulated in NPp53; blue, significantly downregulated in NPp53. (L-N) NP prostate tumors with *Pten* deletion compared to *Nkx3.1-CreERT2* (N) prostates. Red, significantly upregulated in NP; blue, significantly downregulated in NP. Dotted lines indicate thresholds at log2 fold change ≥1.5 and p<0.01. Related to Figure 3.

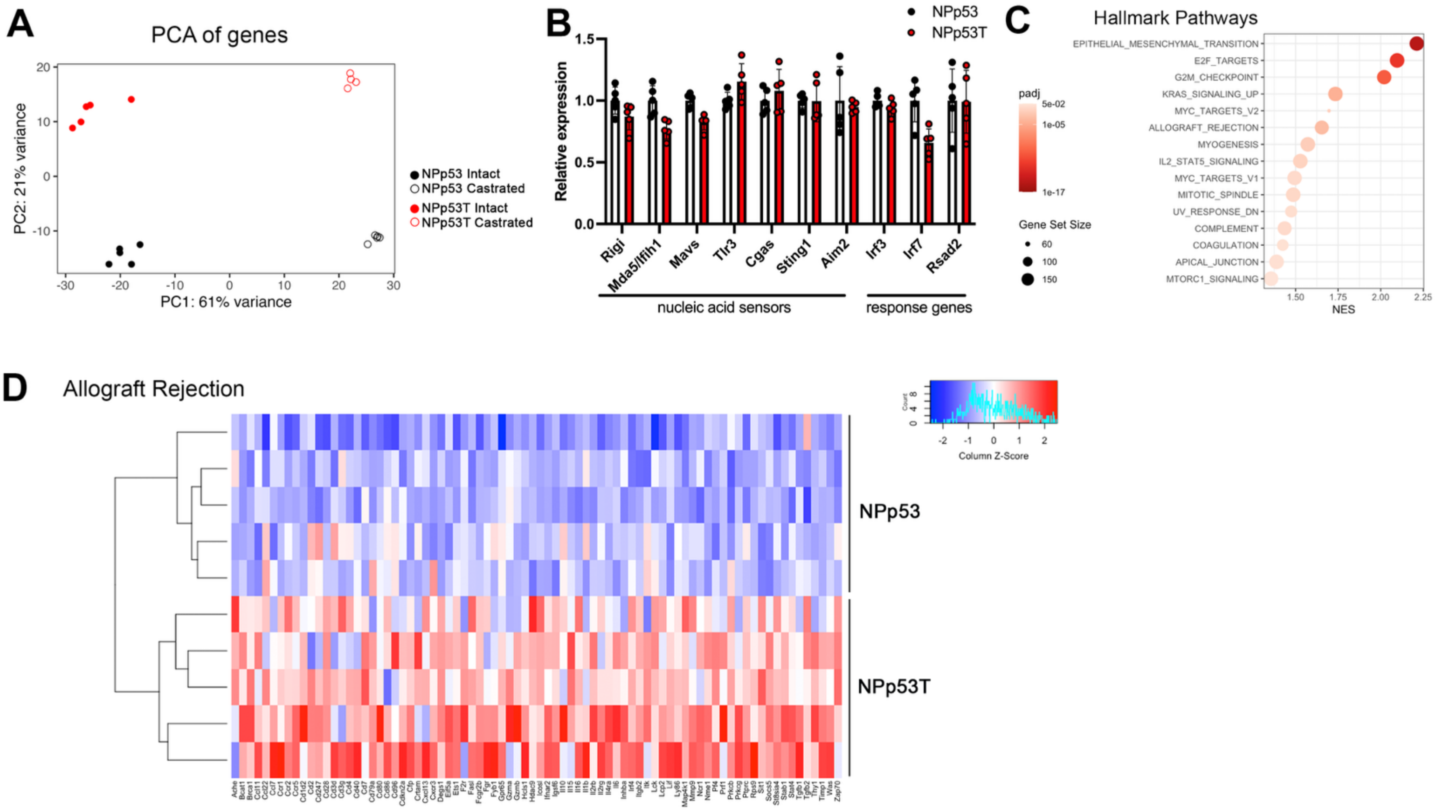

Figure S3. Analysis of gene expression in NPp53T prostate tumors with *Trim28* deletion. (A) Principal components analysis of scaled gene expression in prostate tissues from hormonally intact and castrated mice. n=4 or 5 mice for each genotype. **(B)** Normalized expression of genes involved in viral mimicry response obtained from bulk RNA sequencing of NPp53 and NPp53T tumors. Sample size was n=5 prostates. Error bars represent *s.d.* **(C)** Bubble plot of Hallmark pathways enriched in NPp53T compared to NPp53 prostate tumors from hormonally intact mice. Pathways with the highest normalized enrichment scores (NES) out of 50 well-defined biological processes are shown. **(D)** Heatmap of leading edge genes involved in allograft rejection from Fig. 3I showing differential expression in NPp53T prostate tumors compared to NPp53 prostate tumors. Related to Figure 1, Figure 2 and Figure 3.

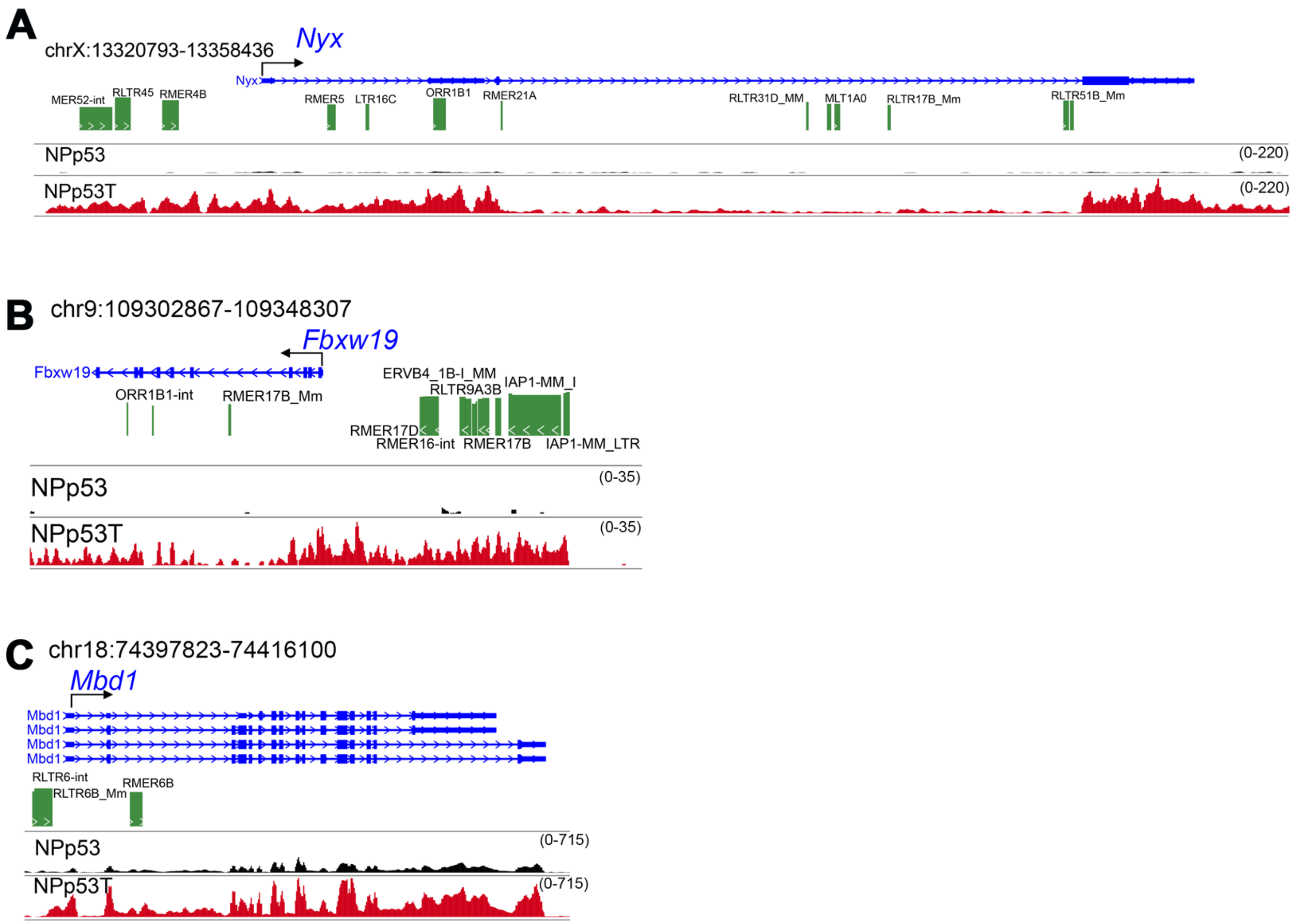

Figure S4. ERVs may promote aberrant gene expression in NPp53T tumors. (A-C) Representative tracks from bulk RNA sequencing of NPp53 (black) and NPp53T (red) prostate tumors (n=5 for each genotype). RefSeq transcripts (blue) and RepeatMasker LTR repeats on the same strand (green) are shown. Related to Figure 4.

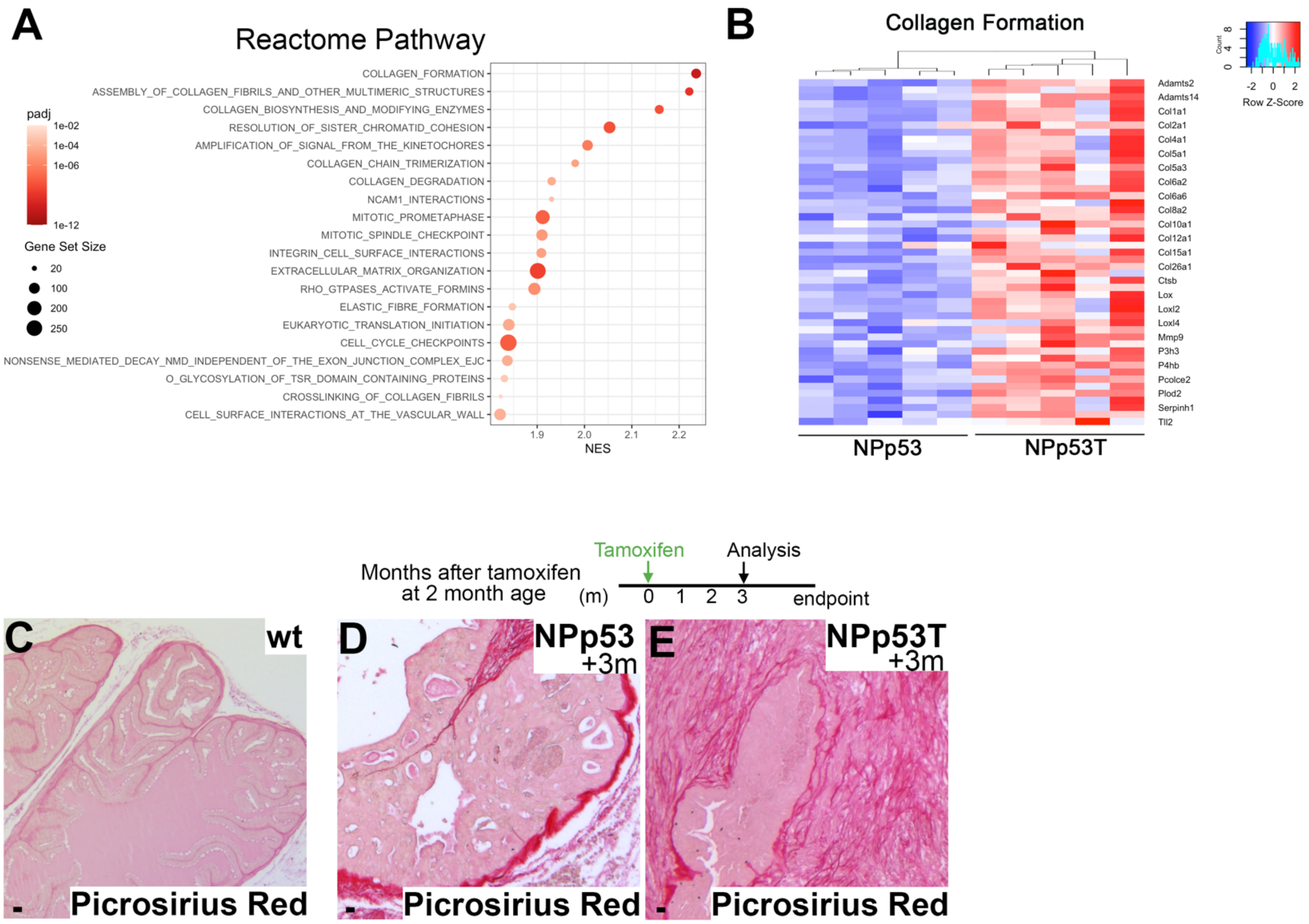

Figure S5. *Trim28* deletion promotes increased collagen formation. (A) Bubble plot of the Reactome pathways with the highest normalized enrichment scores (NES) from gene set enrichment analysis (GSEA) of NPp53T compared to NPp53 prostate tumors (n=5 samples per genotype. 1309 Reactome pathways were analyzed. padj, adjusted p value. **(B)** Heatmap of leading edge genes involved in collagen formation from Fig. 5G showing differential expression in NPp53T prostate tumors compared to NPp53 prostate tumors. **(C-E)** Picrosirius red staining for collagen fibers in (C) 2 month old C57BL/6 wildtype (wt), (D) NPp53 or (E) NPp53T anterior prostates 3 months after tumor induction (n=4-5 prostates for each genotype). Scale bars indicate 50 µm. Related to Figure 5.

Table S1. Primers for qRT-PCR

Target	Forward	Reverse
ERVB2_1A-I_MM chr4:144532663-144533594	ATCGCCAGGCAAATGAATGT	ATACATGGGAGTTTGGGGCA
ERVB5_1-I_MM chr4:144540608-144542116	CACCTGGGCAATTTTCGTGG	TGGCTCACCCAAAATCCTCC
IAPEY3-int chr13:3429445-3433463	GCAGCAGGGTGGATATTTGG	TTAACCCGTCCTGTAACCCC
MER52-int chrX:13321817-13322807	AATGAAGGAGACCCAAACCGA	AAGGACTCAAAGCTGGGACC
RLTR44D chr11:120652159-120652360	AGGTAGAAGCCAGAAGTAGTGA	GGGTTCATGCAGCAACATCT
Gapdh	TGCGACTTCAACAGCAACTC	GCCTCTCTTGCTCAGTGTCC

Table S2. Antibodies

**Antibodies for immunostaining**			
**Antigen**	**Supplier and RRID**	**Species**	**Dilution**
CD3	eBioscience 14-0032-82 (AB_467053)	rat IgG2b	1:100
CD206	Biolegend 141701 (AB_10900263)	rat IgG2a	1:200
dsRNA (J2)	ExAlpha Biologicals, 10010200 (AB_2651015)	mouse IgG2a	1:200
GFP/YFP	Abcam ab13970 (AB_300798)	chicken IgY	1:1000
PTPN22	Invitrogen PA5-118870 (AB_2903370)	rabbit IgG	1:300
**Antibodies for RNA dot blot**			
**Antigen**	**Supplier and RRID**	**Species**	**Dilution**
dsRNA (J2)	ExAlpha Biologicals, 10010200 (AB_2651015)	mouse IgG2a	1:500

Supplementary file 1. Transposable element expression in NPp53T compared to NPp53 prostates.

Supplementary file 2. Gene expression in NPp53T compared to NPp53 prostates.

Supplementary file 3. Gene expression in *Trim28* knockout compared to wildtype cells.
Differential expression in ES cells, neural progenitor cells and liver tissue from GSE41903, GSE45930 and GSE74278 datasets.
